# *Salmonella enterica* induces and subverts the plant immune system

**DOI:** 10.3389/fmicb.2014.00141

**Published:** 2014-04-04

**Authors:** Ana V. García, Heribert Hirt

**Affiliations:** ^1^Unité de Recherche en Génomique Végétale, Unité Mixte de Recherche Institut National de la Recherche Agronomique/Centre National de la Recherche Scientifique/Université Evry Val d’EssonneEvry, France; ^2^Center for Desert Agriculture, King Abdullah University of Science and TechnologyThuwal, Saudi Arabia

**Keywords:** *S. enterica*, flagellin, PAMP-triggered immunity, effector, plants

## Abstract

Infections with *Salmonella enterica* belong to the most prominent causes of food poisoning and infected fruits and vegetables represent important vectors for salmonellosis. Although it was shown that plants raise defense responses against *Salmonella*, these bacteria persist and proliferate in various plant tissues. Recent reports shed light into the molecular interaction between plants and *Salmonella*, highlighting the defense pathways induced and the means used by the bacteria to escape the plant immune system and accomplish colonization. It was recently shown that plants detect *Salmonella* pathogen-associated molecular patterns (PAMPs), such as the flagellin peptide flg22, and activate hallmarks of the defense program known as PAMP-triggered immunity (PTI). Interestingly, certain *Salmonella* strains carry mutations in the flg22 domain triggering PTI, suggesting that a strategy of *Salmonella* is to escape plant detection by mutating PAMP motifs. Another strategy may rely on the type III secretion system (T3SS) as T3SS mutants were found to induce stronger plant defense responses than wild type bacteria. Although *Salmonella* effector delivery into plant cells has not been shown, expression of *Salmonella* effectors in plant tissues shows that these bacteria also possess powerful means to manipulate the plant immune system. Altogether, these data suggest that *Salmonella* triggers PTI in plants and evolved strategies to avoid or subvert plant immunity.

## INTRODUCTION

Several reports have demonstrated that certain human pathogens can colonize plants both at pre- and post-harvest stages, which is the cause of various outbreaks of foodborne human illnesses (Fletcher et al., 2013). These findings have expanded the research interest on so-called human pathogens on plants (HPOPs) as a means to explore and develop new avenues to increase food safety. One important HPOP is the Gram-negative bacterium *Salmonella enterica*, the causative agent of diseases such as gastroenteritis and typhoid fever that every year is responsible of outbreaks related to the consumption of raw fruits and vegetables (Centers for Disease Control and Prevention; http://www.cdc.gov/foodsafety/outbreaks/multistate-outbreaks/outbreaks-list.html). Indeed, non-typhoidal *S. enterica* serovars can be internalized and persist in several plant species. Using GFP-labeled bacteria, it was shown that *S. enterica* can enter plant leaves through natural openings, such as hydathodes in tomato (Gu et al., 2013) and stomata in lettuce (Kroupitski et al., 2009), or by forcing themselves into plant tissues via “weak points” such as lateral root junctions (Cooley et al., 2003). Once in the intercellular space named apoplast, *S. enterica* is safe from regular sanitizer treatments, but in order to persist inside plant tissues it needs to cope with the plant immune system.

Plants lack an adaptive immune system as exists in higher animals but they have multilayered defense mechanisms that resist infection by a large variety of potential pathogenic microorganisms. The first layer of induced defenses is mediated by plasma membrane localized pattern-recognition receptors (PRRs) that detect conserved microbial features termed pathogen- or microbe-associated molecular patterns (PAMPs or MAMPs). Most characterized PRRs possess an extracellular sensing domain, a transmembrane region and an intracellular protein kinase domain that activates a chain of signaling events upon recognition of external molecules (Monaghan and Zipfel, 2012).These signaling events start with the rapid formation of a receptor complex at the plasma membrane, the activation of kinase cascades involving mitogen-activated protein kinases (MAPKs) and the production of reactive oxygen species (ROS) within minutes but also include slower events such as a transcriptional reprogramming and production of the defense hormones salicylic acid (SA) and ethylene (ET; Monaghan and Zipfel, 2012). Altogether these signaling events lead to the so-called pattern-triggered immunity (PTI) which is usually sufficient to stop microbial invasion. Host-adapted pathogens are able to deliver effectors to the apoplast or inside the host cell using delivery systems, such as the bacterial type III secretion system (T3SS), to inactivate PTI components and thereby enable host colonization. A second layer of plant immunity is mediated by intracellular nucleotide-binding leucine-rich repeat receptors (NLR) that recognize the presence or the activity of specific microbial effectors and initiate effector-triggered immunity (ETI). ETI amplifies PTI responses and is normally associated with the appearance of localized cell death lesions known as hypersensitive response (HR; Heidrich et al., 2012). Furthermore, plants need to tailor their defense responses according to the lifestyle of the pathogenic microorganism. Whereas SA-based defenses are efficient to fight biotrophic pathogens that depend on living cells, necrotrophic pathogens that feed on dead tissue induce defense responses mediated by the hormones jasmonic acid (JA) and ET and many other plant hormones further influence the outcome of plant-pathogen interactions (Robert-Seilaniantz et al., 2011).

Several reports have shown that plants are able to detect and mount defense responses to *S. enterica* and recent studies started to shed light on the bacterial features recognized and the plant receptors involved. Here we will review new data concerning the molecular interaction between *S. enterica* and plants, and highlight key aspects of the interaction that are still unclear.

## *Salmonella enterica* INDUCES PTI IN PLANTS

The recognition of *S. enterica* in animals occurs through its O antigen, reflecting variation in the lipopolysaccharide (LPS), and its H antigen, reflecting variation in flagellin, and is essential for activating animal innate immunity (Broz et al., 2012). Most *S. enterica* serovars carry two flagellin-encoding genes, *fliC* and *fljB*, and have the capability of “phase variation” through which *Salmonella* alternate between the expression of the two flagellar genes (Silverman and Simon, 1980). Bacterial flagellin constitutes the best studied PAMP recognition system in plants, whereas LPS perception and induced signaling cascades are less characterized (Zipfel et al., 2004; Sun et al., 2012). In plants, flagellin is recognized through direct binding of a conserved N-terminal domain called flg22 by the LRR receptor kinase FLS2 (flagellin-sensing 2) (Gomez-Gomez and Boller, 2000; Zipfel et al., 2004). Recently, a second domain at the N-terminal region of *Pseudomonas syringae* flagellin, termed flgII-28, was shown to be recognized in certain solanaceous species (Cai et al., 2011; Clarke et al., 2013).The N-terminal region spanning the plant recognized domains are identical in the two flagellin proteins encoded by *S. enterica* serovar *Typhimurium* (*S*. *Typhimurium*) and present some amino acid differences with respect to the sequences shown to be recognized in plants (Garcia et al., 2013; Meng et al., 2013).

**FIGURE 1:**
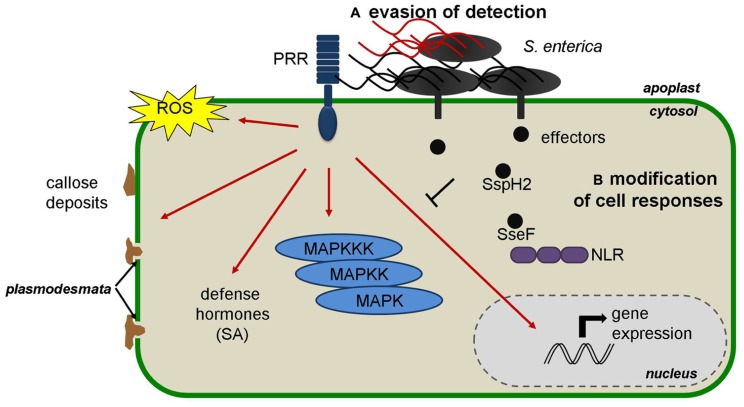
**Representation of the defense responses induced upon *S. enterica* perception in plants and the hypothetical mechanisms that could be used by *S. enterica* to accomplish plant colonization: **(A)** evasion of detection by PRRs through modification of PAMPs such as flagellin and **(B)** delivery of effectors to modify cell responses.** NLR, nucleotide-binding leucine-rich repeat receptor; PRR, pattern-recognition receptor; ROS, reactive oxygen species; SA, Salicylic Acid.

Various studies indicated that *S. enterica* possesses PAMPs that are recognized in plants (**Figure 1**). In a similar approach to that used for the identification of flagellin as a PAMP in plants (Felix et al., 1999), the treatment of tobacco cell cultures with heat-killed *S*. *Typhimurium* elicited rapid ROS accumulation (Shirron and Yaron, 2011). Furthermore, inoculation of *S. enterica* serovars to *Arabidopsis thaliana* seedlings triggered MAPK activation and defense gene expression to a similar extent as that provoked by *P. syringae* inoculation (Schikora et al., 2008, 2011; Garcia et al., 2013). Using a cell death suppression assay Meng et al. (2013) further confirmed that *S. enterica* induces PTI when infiltrated into *Nicotiana benthamiana* leaves. The induction of PTI hallmarks was reduced in *Arabidopsis fls2* mutant seedlings and in *N. benthamiana* leaves silenced for *FLS2* (Garcia et al., 2013; Meng et al., 2013). Furthermore, *S. enterica* flagellin mutants triggered reduced defense responses in *Arabidopsis* and tomato, and colonized *Medicago* spp. to higher numbers compared to wild type bacteria (Iniguez et al., 2005; Garcia et al., 2013; Meng et al., 2013). Together, these results demonstrated that *S. enterica* flagellin is recognized in plants via FLS2. PTI induction was mostly dependent on the *fliC* gene, the most widespread flagellin-encoding gene in *S. enterica* populations (Meng et al., 2013).The *S. enterica* flg22 sequence shows five amino acid changes with respect to the canonical flg22 from *P. aeruginosa* but nevertheless the purified peptide corresponding to the *S. enterica* flg22 sequence (flg22-ST) activated several PTI hallmarks, such as ROS accumulation, callose deposition, growth reduction, and resistance, to a similar extent as *Pseudomonas* flg22 (Garcia et al., 2013; Meng et al., 2013). Interestingly, a recent report demonstrated that *S. Typhimurium* triggers stomatal closure, another PTI-related defense response that limits pathogen entry into the apoplast (Roy et al., 2013). Bacteria-induced stomatal closure is largely mediated by FLS2-mediated recognition of flagellin (Melotto et al., 2006; Zeng and He, 2010). It is therefore intriguing that *S. Typhimurium* treatment of *Arabidopsis* and lettuce leaves triggered reduced stomatal closure as compared with *Escherichia coli* (Roy et al., 2013), even if both bacteria carry the same flg22 sequence (Garcia et al., 2013). Furthermore, *Salmonella* treated leaves showed stronger stomatal reopening 4 h after bacterial inoculation (Roy et al., 2013). These differences could be due to the action of effector molecules or phytotoxins of *Salmonella* that interfere with plant stomatal immunity and that may be absent or less efficient in *E. coli*. Otherwise, it is also possible that the stronger responses triggered by *E. coli* can be attributed to the recognition of other PAMPs such as LPS, which also trigger stomatal closure (Melotto et al., 2006).

Interestingly, previous studies suggested the existence of a different flg22 sequence in strains of *S. enterica* serovar Senftenberg that is more divergent from the canonical flg22 (Tankouo-Sandjong et al., 2008; Berger et al., 2011). The corresponding flg22 peptide (flg22-SS) displayed reduced PTI activity in *Arabidopsis* and therefore suggests that certain *S. enterica* strains may have evolved divergent flagellin sequences to avoid plant recognition (Garcia et al., 2013; **Figure 1**). Other reports have already shown the existence of intra-species variation in the flg22-encoding regions of the *fliC* genes of *P. syringae* and *Xanthomonas campestris* (Sun et al., 2006; Cai et al., 2011; Clarke et al., 2013), providing evidence that PAMPs are less conserved than usually assumed and can evolve to avoid activation of plant defense responses.

The results gathered so far also indicate that other *S. enterica* PAMPs besides flagellin are recognized in plants, as certain PTI activation was still observed in the *Arabidopsis fls2* mutant or after inoculation with *S. enterica* flagellin mutants (Garcia et al., 2013; Meng et al., 2013). In this line, it was reported that *S. enterica* strains carrying the O antigen 1,3,19 induce leaf chlorosis and wilting when infiltrated into *Arabidopsis* leaves (Berger et al., 2011). Furthermore, purified LPS from *S. Typhimurium* (carrying another O antigen) induced ROS accumulation in tobacco (Shirron and Yaron, 2011). On the contrary, LPS purified from other serovars did not activate PTI when infiltrated into *N. benthamiana* leaves or induce ROS in tomato (Meng et al., 2013). This suggests that either a specific O antigen is recognized only by a reduced group of plant species or that other molecule(s) in the strains carrying the O antigen 1,3,19 is recognized in *Arabidopsis*. Synthetic peptides representing conserved regions of cold shock proteins (CSPs) were also inactive in *N. benthamiana* but presented a mild activity in ROS assays in tomato (Meng et al., 2013). Altogether these results indicated that the flagellin flg22 domain is the most prominent PAMP from *S. enterica* recognized in plants. Other PAMPs such as CSPs and flgII-28 seem to contribute to PTI activation to a lesser extent (Meng et al., 2013) and the recognition of *S. enterica* LPS in plants is still unclear.

Data suggests that defense hormones are also involved in the interaction between *S. enterica* and plants. Indeed, Arabidopsis mutants or transgenic lines affected in SA as well as in JA and ET signaling pathways showed increased *S. enterica* colonization (Iniguez et al., 2005; Schikora et al., 2008). Furthermore, pre-treatment with the ET precursor ACC (1-Aminocyclopropane-1-carboxylic acid) reduced the colonization of *Arabidopsis* and alfalfa (*Medicago sativa*) roots by *S. Typhimurium*, as well as by other bacterial endophytes (Iniguez et al., 2005). Recently, it was shown that treatment of *Arabidopsis* seedlings with *S. Typhimurium* wild type and *prgH-* mutant leads to a mild but significant increase in SA accumulation and the reprogramming of several marker genes of the SA pathway (Garcia et al., 2013; **Figure 1**). Phytohormones can mold plant–microbe interactions in a positive or negative way depending on the pathogen, which in turn can be exploited by microbes to induce susceptibility (Robert-Seilaniantz et al., 2011). Therefore, whereas it is clear that defense hormones play a role in the interaction between *S. enterica* and plants, further information is needed to depict which hormones contribute to resistance and if certain hormones are induced by *S. enterica* to increase host susceptibility.

## A ROLE FOR *Salmonella enterica* EFFECTORS IN PLANT TISSUES

*Salmonella* has two pathogenicity islands, SPI-1 (*Salmonella* pathogenicity island 1) and SPI-2, which encode a T3SS (T3SS-1 and T3SS-2) and a suite of effectors. These two secretion systems contribute to different stages of the animal infection process: while the T3SS-1 is expressed at the extracellular stage the T3SS-2 is induced after internalization into animal cells. More than 30 Salmonella effectors have been studied and shown to play important functions for virulence in animal cells by manipulating diverse host cell functions (McGhie et al., 2009). Recently, *Salmonella* T3SSs and effectors were proposed to contribute to the plant colonization process. *S. Typhimurium* mutants in T3SS-1 and T3SS-2 induced stronger cell death and chlorosis symptoms and proliferated to lower levels in *Arabidopsis* leaves (Schikora et al., 2011). Furthermore, the *S. Typhimurium prgH-* mutant, carrying a mutation in a structural component of the SPI-1-encoded T3SS needle complex, triggered enhanced expression of defense genes in *Arabidopsis* seedlings (Schikora et al., 2011; Garcia et al., 2013). Besides, the *S. Typhimurium invA-* mutant, another T3SS-1 defective mutant, induced stronger ROS accumulation than wild type bacteria in tobacco BY-2 cells (Shirron and Yaron, 2011). These results suggested that *S. enterica* effector delivery may be important for plant colonization by dampening the plant immune system (**Figure 1**). On the contrary, the initial colonization of alfalfa roots was higher when using *S. enterica* mutants in the SPI-1-encoded *spas* and *sipB* genes, with defects in effector delivery, which indicates the possibility that certain SPI-1 encoded molecules are recognized and trigger defense responses in this host (Iniguez et al., 2005). Finally, it was recently reported that the *S. Typhimurium prgH-* mutant can multiply to similar levels as the wild type strain in tomato leaves, which led the authors to conclude that SPI-1 is not important for tomato colonization and that tomato plants do not recognize SPI-1 products (Meng et al., 2013). The different results obtained in different plant species are intriguing and may point to different mechanisms of interaction.

So far no study has demonstrated the *Salmonella*-mediated delivery of effectors into plant tissues but two recent studies suggest that *S. enterica* effectors are functional in plant cells. Ustun et al. (2012) used *Agrobacterium tumefaciens* to monitor the effect of several *S. Typhimurium* effectors when expressed in *N. benthamiana* and identified the SPI-2-encoded effector SseF as an inducer of HR-like cell death lesions. The appearance of cell death lesions was specific and accompanied by the upregulation of several cell death and defense-related genes. Furthermore, SseF also triggered HR-cell death and ETI when delivered into *N. benthamiana* leaves in a T3SS-dependent manner by inoculation with *X. campestris* pv. *vesicatoria*. Interestingly, the SseF-triggered cell death was compromised by silencing the *N. benthamiana* genes coding for the co-chaperone SGT1 (suppressor of G2 allele of skp1) and the plasma-membrane localized protein NDR1 (non-race-specific disease resistance 1), two proteins normally required for resistance mediated by NLRs carrying an N-terminal coiled-coil domain (Austin et al., 2002; Knepper et al., 2011; Ustun et al., 2012). Altogether, these data suggested that the *S. Typhimurium* SseF effector is recognized by an NLR in *N. benthamiana*. Recently, another SPI-2-encoded effector, SspH2, was proposed to perform functions conserved in plant and animal cells (Bhavsar et al., 2013). It was demonstrated that SspH2, an E3 ubiquitin ligase, interacts with and increases the activity of animal and plant SGT1 proteins, which in turn increases NLR-mediated cell death (Bhavsar et al., 2013). The functional significance of this interaction is unclear, but it demonstrates that *S. enterica* effectors are able to manipulate plant and animal immune components (**Figure 1**).

## FURTHER HOST ADAPTATION MECHANISMS

The colonization of plants can also lead to molecular and phenotypical changes in *S. enterica* cells, which can help us to identify the determining factors governing the interaction between *S. enterica* and plants. The genome sequences of several *S*. *enterica* serovars are known and transcriptome analyzes have successfully been used to assess gene expression changes in environmental conditions associated to the animal infection processes (Hébrard et al., 2011; Kroger et al., 2013). Recently, the first transcriptome analysis of *S. enterica* cells in contact with plants was reported (Goudeau et al., 2013). Given that *S. enterica* proliferation levels are higher in soft-rotted tissues, the authors analyzed the transcriptional changes in *S. Typhimurium* upon inoculation of *Dickeya dadantii*-macerated cilantro (*Coriandrum sativum*) and lettuce (*Lactuca sativa*) leaves. This analysis revealed significant changes in gene expression that includes the upregulation of various nutritional and metabolic pathways, suggesting that *S. enterica* reacts to and benefits from the enhanced nutrient availability in the soft-rotted leaves (Goudeau et al., 2013). It is still intriguing to know how the plant environment impacts on the expression of other virulence related genes, such as the *Salmonella* pathogenicity islands and the encoded T3SS and effectors. For instance, SPI-1 genes are known to be induced by high osmolarity, low oxygen levels and short chain fatty acids (Hautefort et al., 2003) and using a promoter-reporter fusion, it was recently shown that the SPI-1 gene *prgH* is expressed when in contact with *Arabidopsis* root cells (Garcia et al., 2013).

Furthermore, genetic screens monitoring the efficiency of *S. enterica* mutants in different steps of the plant colonization process have proved useful to identify bacterial genes that are important for the interaction with plants (Barak et al., 2005, 2009). These analyzes pointed to the synthesis of cellulose and aggregative fimbriae (curli), involved in the so-called “rdar” (red dry and rough) phenotype, as being important for plant attachment. Interestingly, several non-rdar mutants have been recovered from produce-related disease outbreaks (Brandl et al., 2013). Furthermore, *S. Typhimurium* non-rdar mutants originating by sequential passages through tomatoes showed enhanced fitness in plants as compared to the parental strain, despite being less competitive on common laboratory media (Zaragoza et al., 2012). Altogether these results indicate that *S. enterica* has the ability to rapidly evolve and adapt to specific host environments such as the hostile plant apoplast. Further gene expression analyzes and mutant screens will allow us to gain insight into the virulence mechanisms used by *S. enterica* to colonize and survive in plant tissues.

## PERSPECTIVES

In the past few years, the research on HPOPs has expanded and demonstrated that *S. enterica* serovars are able to colonize and persist in different plant tissues and species. These studies have also suggested that the ability to colonize different hosts is variable and governed by various genetic and environmental factors. A few studies reported varied proliferation levels (one to five logs) of *S. Typhimurium* inside plant tissues (Cooley et al., 2003; Schikora et al., 2008, 2011; Barak et al., 2011; Garcia et al., 2013; Meng et al., 2013) and evidence suggests that *S. enterica* titers inside the plant apoplast are highly dependent on the environmental conditions (Cooley et al., 2003; Roy et al., 2013). It is clear that the increased interest in HPOPs will reveal the mechanisms used by these microorganisms to exploit plants as secondary hosts and should help to develop strategies to reduce *S. enterica* populations in plants and thereby disease outbreaks.

## Conflict of Interest Statement

The authors declare that the research was conducted in the absence of any commercial or financial relationships that could be construed as a potential conflict of interest.
